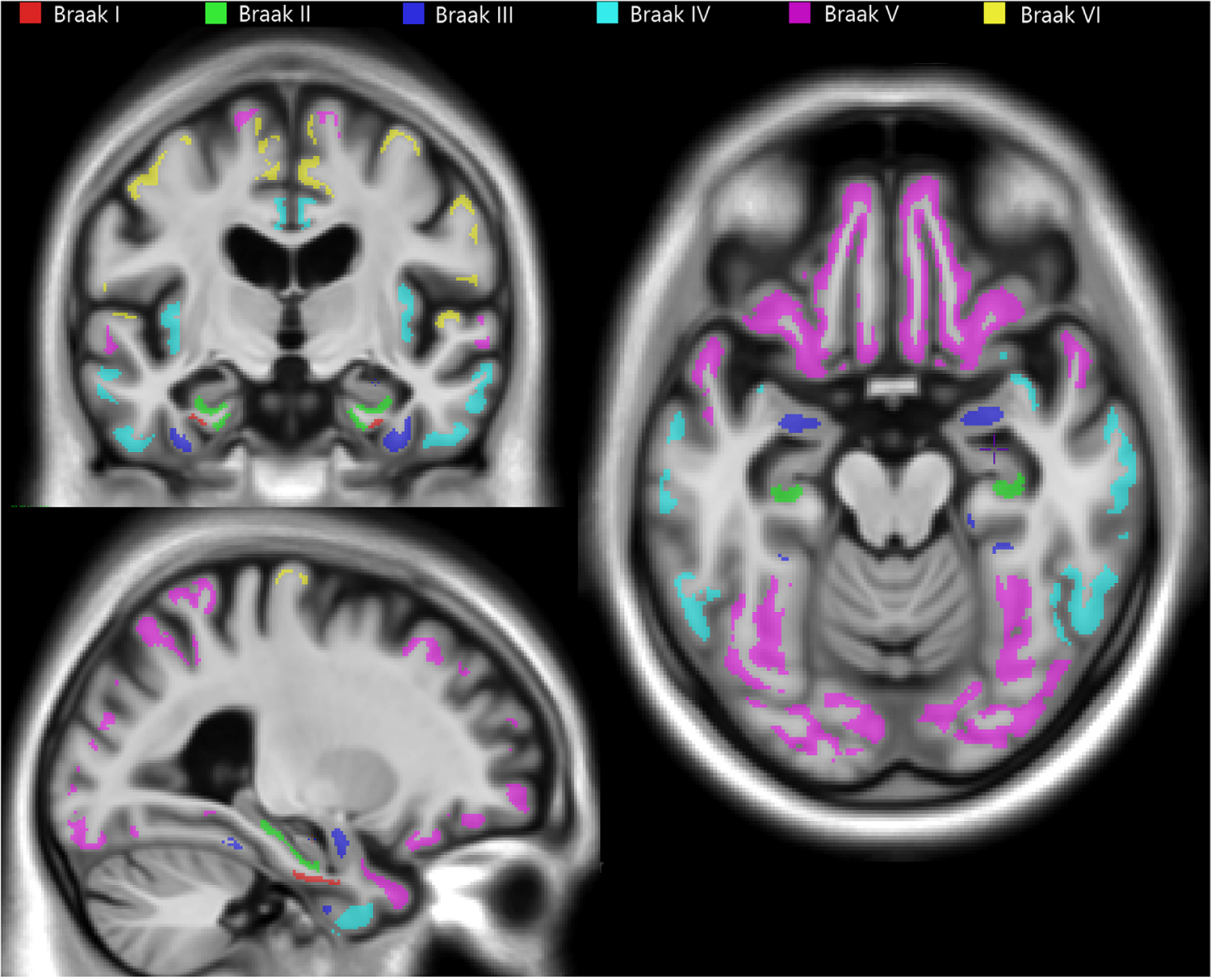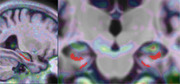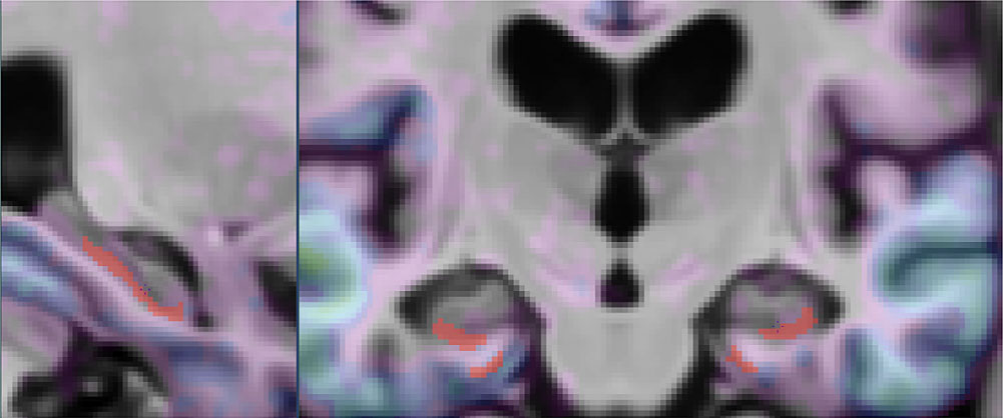# Refining early tau‐PET staging using CA1 and prosubiculum rather than the whole hippocampus

**DOI:** 10.1002/alz70856_107689

**Published:** 2026-01-09

**Authors:** Etienne Aumont, Seyyed Ali Hosseini, Brandon J Hall, Tevy Chan, Lydia Trudel, Jaime Fernandez Arias, Joseph Therriault, Nesrine Rahmouni, Gleb Bezgin, Jenna Stevenson, Stijn Servaes, Arthur C. Macedo, Serge Gauthier, Pedro Rosa‐Neto

**Affiliations:** ^1^ McGill University, Montreal, QC, Canada; ^2^ Montreal Neurological Institute, Montreal, QC, Canada

## Abstract

**Background:**

Early tau positron emission tomography (PET) regions of interest (ROIs) either consider the whole hippocampus or wholly excludes it. However, only the cornu ammonis 1 (CA1) and prosubiculum are specifically afflicted by tau pathology in histopathological examinations (1). In this study, we propose a novel approach that aligns with the known distribution of tau in the hippocampus compared with the traditional, whole hippocampus approach.

**Method:**

We quantified the tau neurofibrillary tangle distribution using [^18^F]MK6240 in 842 images from 432 participants aged 55 and over. Tau staging was established using ROI thresholds based on 2.5 standard deviations above 40 young adults (aged 18‐25). Participants were classified as discordant if they met the threshold for a given stage without fulfilling the criteria for a previous stage. We generated two alternative Braak stages based on 1) the classical whole hippocampus approach; 2) a refined Braak II ROI that specifically includes CA1 and prosubiculum (Figure 1). Cases with inconsistent stages between both schemes were visually inspected.

**Result:**

The CA1 and prosubiculum staging resulted in 15 (1.78%) discordant cases, 65% less than the whole hippocampus staging (44, 5.22%). Of these 44 discordant cases, 23 were stage II positive in the whole hippocampus scheme, but negative in the CA1 and prosubiculum scheme. Visual inspection confirmed these as false positives driven by off‐target binding in the choroid plexus. This off‐target binding did not affect the CA1 and prosubiculum ROI (Figure 2). Seven other discordant cases were considered hippocampal‐sparing using the whole hippocampus scheme despite showing significant tau signal specifically in CA1. The CA1 and prosubiculum ROI was able to capture this signal (Figure 3).

**Conclusion:**

Limiting hippocampal tau quantification to the CA1 and prosubiculum improved the accuracy of tau quantification. This improvement was due to a reduction in false positives due to choroid plexus off‐target binding and an improved sensitivity to CA1‐specific tau signal. We recommend adopting this approach to improve the detection of tau pathology using tau‐PET.